# PAQR5 Expression Is Suppressed by TGFβ1 and Associated With a Poor Survival Outcome in Renal Clear Cell Carcinoma

**DOI:** 10.3389/fonc.2021.827344

**Published:** 2022-01-20

**Authors:** Chang Tao, Wang Liu, Xiang Yan, Min Yang, Si Yao, Qiang Shu, Benyi Li, Runzhi Zhu

**Affiliations:** ^1^ National Clinical Research Center for Child Health, The Children’s Hospital, Zhejiang University School of Medicine, Hangzhou, China; ^2^ Department of Urology, The University of Kansas Medical Center, Kansas City, KS, United States; ^3^ Cancer Center, Zhejiang University, Hangzhou, China

**Keywords:** PAQR5, TGFβ1, renal cancer, patient survival, disease progression

## Abstract

**Background:**

Renal cell carcinoma (RCC) was sex-hormone responsive, and clinical trials using progesterone significantly reduced the incidence of distal metastasis after radical nephrectomy. Recently membrane-bound progesterone receptors (mPRs) were discovered to mediate the non-genomic effect of progesterone. Aberrant expressions of these mPRs were reported in human breast, ovarian, urinary bladder, brain, uterine, and prostate cancers. However, their expression profiles in RCC are yet to be assessed.

**Methods:**

Multiple datasets from RNA sequencing (RNA-seq), cDNA microarray, and proteomic analysis were used to compare gene expression between cancerous and normal kidney tissues. Immunohistochemistry was conducted to examine protein expression in kidney tissues. Promoter methylation levels were assessed for correlation analysis with gene expression.

**Results:**

Of the seven membrane-bound progesterone receptor genes, the progestin and adipoQ receptor-5 (PAQR5) gene is predominantly expressed in normal kidney tissue but was significantly downregulated in RCC tissues. PAQR5 downregulation correlated with tumor stage, cancer grade, lymph node invasion, and distal metastasis only in clear cell RCC (ccRCC) tissues. PAQR5 downregulation was associated with an increased promoter DNA methylation and a poor survival outcome in ccRCC patients. In addition, PAQR5 expression inversely correlated with transforming growth factor beta-1 (TGFB1) expression, and TGFβ1 treatment significantly reduced PAQR5 gene expression.

**Conclusion:**

PAQR5 is a novel prognostic biomarker in ccRCC and is negatively regulated by the TGFβ1 pathway.

## Introduction

Renal cell carcinoma (RCC) is the most common kidney cancer, and it is derived from renal tubular cells. Among the 15 histological subtypes of RCC, clear cell RCC (ccRCC) is the most common, followed by papillary RCC (pRCC) and chromophobe RCC (ChRCC). Although localized RCC cases have a relatively good prognosis with treatment with the 5-year survival rate of 74-81% for Stage 1-2 patients, metastatic diseases with Stage 3-4 at diagnosis are suffering a poor 5-year survival rate of only 53% (1). Therefore, novel biomarkers are urgently needed to understand the mechanisms leading to tumor progression and to serve as therapeutic biomarkers for better disease management.

RCC tumors have been considered as sex-hormone responsive based on clinical observation and pathological analysis (2, 3) [reviewed in ref (4)]. The sex hormone binding sites for estrogen, androgen, and progesterone were confirmed in RCC tissues (3). Expression of the receptor proteins, including the estrogen receptor (ER), progesterone receptor (PGR), and androgen receptor (AR), were observed in 30-40% of ccRCC tissues (5). However, a significantly low level of progesterone, but not estradiol-17-β (E_2_), was observed in RCC tissues compared to normal kidney tissues (6). Consistently, clinical trials using progesterone (Medroxyprogesterone Acetate) as a postoperative prophylactic therapy significantly reduced the incidence (10% in treatment groups *vs.* 35% in the control group) of distal metastasis three years after radical nephrectomy for localized patients (7). In contrast, anti-AR flutamide had no clinical benefit in disseminated RCC patients (8). These studies suggest that progesterone has a suppressive function in RCC progression.

In last two decades, a group of membrane-bound progesterone receptors (mPRs) were identified in human reproductive and neuronal tissues to mediate the non-genomic effect of progesterone (9, 10). There are two types of mPR proteins, the Class II progesterone and adipoQ receptor (PAQR) family (10) and the heme-binding protein family termed as progesterone receptor membrane component-1/2 (PGRMC1/2) (11). While most of the literature reports on these mPRs focused on their function in reproductive and neuronal biology, aberrant expressions of these mPRs were also shown in other types of human cancers, including breast, ovarian, urinary bladder, brain, uterine, and prostate (10, 12–17). In human endometrial cancers, increased PAQR5 expression was associated with a favorable patient prognosis (12), whereas increased PAQR6 expression was associated with a poor prognosis in prostate cancers (13). However, there is a lack of research on these mPR genes in human kidney cancers. In this study we conducted a comprehensive analysis of mPR gene expression profiles using multiple datasets from RNA-seq, cDNA microarray, and proteomic analysis. Our research revealed that PAQR5 expression was significantly reduced in RCC tissues but only correlated with disease progression and survival outcomes in ccRCC patients. Our study suggests that PAQR5 downregulation was associated with promoter hypermethylation and TGFB1 gene upregulation in ccRCC tissues.

## Materials and Methods

### Gene Expression Profiles in Normal and Cancerous Tissues

The expression profiles of PAQR5/6/7/8/9, PGR, and PGRMC1/2 genes in normal organs or tissues were assessed using the cDNA microarray datasets (NCBI GSE3526) on the Oncomine platform (18, 19). These datasets were generated with 353 normal tissues on Affymetrix U133 Plus 2.0 microarrays that measured 19,574 genes with 54,675 reporters. A total of 65 primary human organs were utilized in comparison.

The RNA-seq datasets derived from The Cancer Genome Atlas (TCGA) project (20) were used as the primary approach to compare gene expression between normal and cancerous kidney tissues. These TCGA datasets for kidney cancers contained three subtypes of RCC tissues, chRCC (KICH), ccRCC (KIRC), and pRCC (KIRP).

### Immunohistochemistry for PAQR5/7 Expression in Normal and ccRCC Tissues

Tissue microarray slides containing 42 ccRCC and 10 normal kidney sections were purchased from Novus Biologicals (Centennial, CO). After deparaffinization and hydration, tissue slides were treated with 3% H_2_O_2_ for 15 minutes and then blocked with 5% bovine serum albumin (BSA) in tris-buffered saline (TBS) and tween-20 (TBS-T) for 60 minutes. The primary antibody for PAQR5 protein was derived from LSBio (Catalog LSC413057/413055) and used at 1:200 dilution in 5% BSA/TBS-T overnight at 4C with agitation. The immune signals were visualized using the DAKO LSAB2 kit obtained from Agilent (Santa Clara, CA). The immunosignal index was calculated by multiplying the immune density (weak = 1, moderate = 2, strong = 3) with the percentage positivity, as described in our previous report (21).

### Protein Expression Profiles in Normal and Cancerous Tissues

Protein expression in ccRCC and normal kidney tissues was assessed using the proteomic datasets derived for the Clinical Proteomic Tumor Analysis Consortium (CPTAC) (22). Proteomic profiles were available for selective proteins from 110 ccRCC tissues and 84 normal kidney tissues on the UALCAN platform (23). A z-score value was used to present the relative level of protein expression.

### Gene Expression and DNA Methylation Analyses in Patients During Disease Progression

Gene expression analyses at the mRNA level in RCC patients were conducted using the TCGA datasets. Correlation analyses between gene expression levels and PAQR5 promoter DNA methylation were performed using the HM450 methylation data derived from the TCGA project.

### TGFβ1 Treatment in Immortalized Ovarian Surface Epithelial Cells

The microarray dataset (NCBI GDS2975) was used for assessing the effect of TGFβ1 on PAQR gene expression. This dataset was generated on human immortalized ovarian surface epithelial (IOSE) cells as described (24). Briefly, immortalized ovarian surface epithelial (IOSE) cells were treated with TGFβ1 (Millipore-Sigma, St Louis, MO) at 10 ng/ml concentration for 0, 3, 6, 12 hours. Total RNAs from IOSE cells were isolated using the TRIzol reagent (Invitrogen, Waltham, MA) and were subjected to cDNA microarray analysis using the Human Genome U133A plus 2.0 GeneChip Oligonucleotide Array (Affymetrix, Santa Clara, CA).

### Assessment of Patient Survival Outcomes

Patient survival outcomes, including overall survival, disease-specific survival, and progression-free interval, were assessed using the Kaplan-Meier curve approach (25). Patients were stratified into high or low expression groups using the minimum p-value approach (26). The significance of the hazard ratio was statistically analyzed using the Log-rank test. A nomogram was constructed using the R-rms package (version 6.2-0) and the survival package (version 3.2-10) based on PAQR5 expression and patient clinicopathological parameters, as described (27).

### Data Presentation and Statistical Analysis

Quantitative data for gene expression at the mRNA and protein levels were presented as the MEAN with the SEM (standard error of the mean). Differences among multiple groups were analyzed using the statistical methods described in the figure legend. Microscopic images from immunohistochemistry were representative of normal and cancerous kidney tissues. The semi-quantitative intensity of the immunosignals was analyzed using the Student *t*-test as described (21).

## Results

### PAQR Gene Expression Is Significantly Altered in RCC Tissues

We first surveyed the expression patterns of PAQR, PGR, and PGRMC genes in normal tissues using a cDNA microarray dataset generated from 65 types of normal tissues (19). As shown in **Supplementary Figure S1**, the PAQR5 gene was expressed at a relatively higher level in renal tissues (**Supplementary Figure S1A**). In contrast, PAQR6/7/8 genes were ubiquitously expressed in all tested tissue types with variable levels (**Supplementary Figures S1B–D**). PAQR9 was highly expressed in the liver, heart, and testis (**Supplementary Figure S1E**), and PGR expression was mainly in the gonads and female reproductive tissues (**Supplementary Figure S1F**), as expected. Similar to PAQR6/7/8 genes, PGRMC1/2 were ubiquitously expressed in all types of tissues (**Supplementary Figures S1G, H**). These data indicate that the PAQR5 gene might have a critical role in the kidney than in other organs.

**Figure 1:**
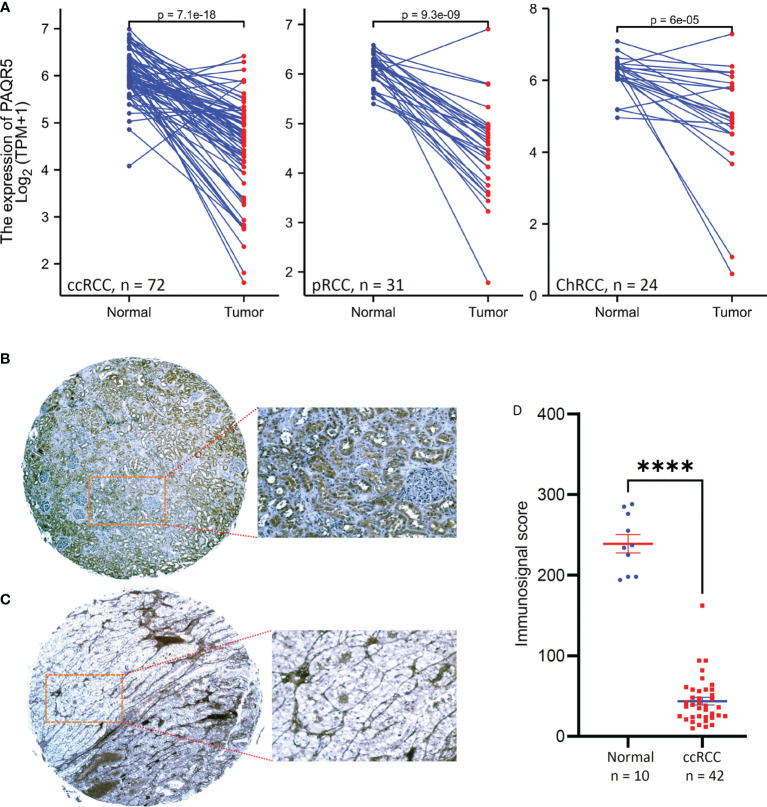
PAQR5 expression in RCC tissues. **(A)** Pair-wise comparison was conducted using the RNA-seq datasets from the TCGA project. The p-values were derived from Wilcoxon signed-rank test. **(B, C)** Representative microscopic images were taken from anti-PAQR5 immunohistochemistry on the normal kidney **(B)** and ccRCC tissue **(C)** sections. Magnification x 200. **(D)** Semi-quantitative data from immunohistochemistry staining were compared between normal kidney and ccRCC tissues. The error bars indicate the MEAN and SEM. The Quadro asterisk indicates a significant difference (Student *t*-test, p < 0.0001).

Since the PAQR5 gene is highly expressed in normal kidney tissue, we then focused our assessment on PAQR5 expression in kidney cancers using the RNA-seq dataset from the TCGA project. There were case-matched normal and cancerous tissue pairs from renal cell carcinoma patients, and a pair-wise comparison was conducted to analyze PAQR expression. Our results showed a significant reduction of PAQR5 expression in all three types of RCC tissues compared to the normal counterparts (**Figure 1A**), except few cases showed increased expression in RCC tissues. In addition to kidney cancers, other human cancers also showed significant alterations in PAQR5 gene expression, including upregulation in cancers from the breast, bile duct, liver, and uterine corpus, and downregulation in cancers from the colon, brain, lung, adrenal gland, prostate, rectal, and thyroid organs (**Supplementary Figure S2**).

To verify PAQR5 downregulation, we examined PAQR5 protein expression in ccRCC tissues using an immunohistochemistry approach. As shown in **Figure 1B**, PAQR5 protein was highly expressed in normal kidney tissues, exclusively in the renal tubular cells but not in the glomerulus cells. In contrast, PAQR5 protein was only expressed in stromal cells of ccRCC tissues and were almost lost in cancer cells (**Figure 1C**). Summarized immunosignal data showed that PAQR5 protein expression levels were significantly reduced in ccRCC tissues compared to normal kidney tissues (**Figure 1D**). These results demonstrate that PAQR5 expression was significantly downregulated in RCC tissues.

### PAQR5 Downregulation Is Associated With Tumor Progression in ccRCC Tissues

To evaluate the clinical significance of PAQR5 gene downregulation, we analyzed the association between PAQR5 expression and clinicopathological parameters using the TCGA RNA-seq dataset. RCC patients were divided into PAQR5^low^ and PAQR5^high^ subgroups at the median levels of PAQR5 expression. The clinicopathological parameters included TNM category, pathological and clinical stages, patient gender, overall and disease-specific survival (DSS) status, and progression-free interval (PFI). Our analysis revealed that PAQR5 expression was significantly associated with all the clinicopathological parameters in ccRCC cases (**Table 1**) but not in pRCC patients (**Supplementary Table S1**). In ChRCC patients, PAQR5 expression was only significantly associated with tumor stage but not with other parameters (**Supplementary Table S2**). These results indicate that PAQR5 downregulation is only related to ccRCC disease progression.

**Table 1:** Association of PAQR5 expression with clinicopathological parameters in ccRCC patients.

Characteristic	Low PAQR5	High PAQR5	p	statistic	method
n	269	270			
T stage, n (%)			<0.001	45.84	Chisq.test
T1	101 (18.7%)	177 (32.8%)			
T2	43 (8%)	28 (5.2%)			
T3	115 (21.3%)	64 (11.9%)			
T4	10 (1.9%)	1 (0.2%)			
N stage, n (%)			0.023	5.18	Chisq.test
N0	117 (45.5%)	124 (48.2%)			
N1	13 (5.1%)	3 (1.2%)			
M stage, n (%)			<0.001	14.86	Chisq.test
M0	197 (38.9%)	231 (45.7%)			
M1	55 (10.9%)	23 (4.5%)			
Pathologic stage, n (%)			<0.001	44.51	Chisq.test
Stage I	98 (18.3%)	174 (32.5%)			
Stage II	34 (6.3%)	25 (4.7%)			
Stage III	77 (14.4%)	46 (8.6%)			
Stage IV	58 (10.8%)	24 (4.5%)			
Gender, n (%)			0.016	5.83	Chisq.test
Female	79 (14.7%)	107 (19.9%)			
Male	190 (35.3%)	163 (30.2%)			
Histologic grade, n (%)			<0.001	40	Chisq.test
G1	4 (0.8%)	10 (1.9%)			
G2	96 (18.1%)	139 (26.2%)			
G3	106 (20%)	101 (19%)			
G4	61 (11.5%)	14 (2.6%)			
OS event, n (%)			<0.001	37.44	Chisq.test
Alive	149 (27.6%)	217 (40.3%)			
Dead	120 (22.3%)	53 (9.8%)			
DSS event, n (%)			<0.001	50.43	Chisq.test
Alive	175 (33.1%)	245 (46.4%)			
Dead	87 (16.5%)	21 (4%)			
PFI event, n (%)			<0.001	46.29	Chisq.test
Alive	152 (28.2%)	226 (41.9%)			
Dead	117 (21.7%)	44 (8.2%)			
Age, median (IQR)	61 (53, 70)	60 (51, 69)	0.310	38151	Wilcoxon

**Figure 2:**
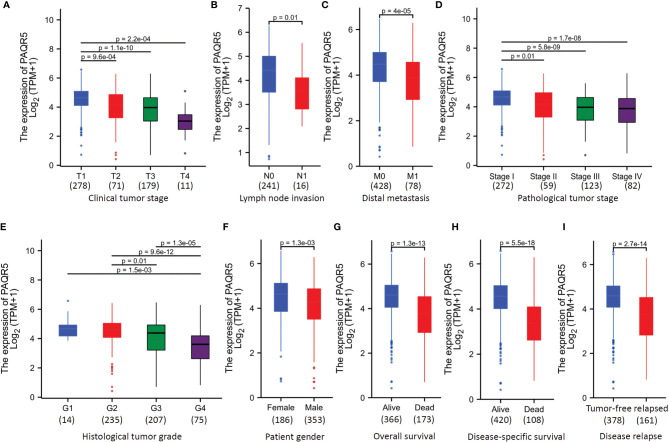
PAQR5 expression is inversely associated with disease progression in ccRCC patients. **(A–I)** The RNAseq dataset from the TCGA project was used to compare different clinicopathological parameters. Multiple group comparison (panel **A, D, E**) was conducted using the Kruskal-Wallis test followed by Bonferroni modification and Dunn’s test. Two group comparison (panel **B, C, F–I**) was performed using the Wilcoxon rank-sum test. Case numbers in each sub-group were listed at the bottom of each panel.

To verify the significance of PAQR5 association with disease progression in ccRCC patients, we compared PAQR5 expression levels with different clinicopathological parameters. As shown in **Figures 2A–E**, PAQR5 expression levels were significantly lower in patients with late-stage tumors (T3-T4 *vs.* T1-2), lymph node invasive tumors (N1 *vs.* N0), distal metastasis (M1 *vs.* M0), late clinical stage tumors (pT3-4 *vs.* pT1-2), and high-grade tumors (G3-4 *vs.* G1-2). Interestingly, male patients also showed a significantly lower PAQR5 expression than female patients (**Figure 2F**). In addition, patients who were deceased or relapsed also displayed a substantially lower level of PAQR5 expression (**Figures 2G–I**). These data demonstrate that PAQR5 downregulation was associated with ccRCC disease progression and metastasis, as well as poor survival status.

### PAQR5 Downregulation Correlates With High Promoter Methylation in ccRCC Tissues

**Figure 3:**
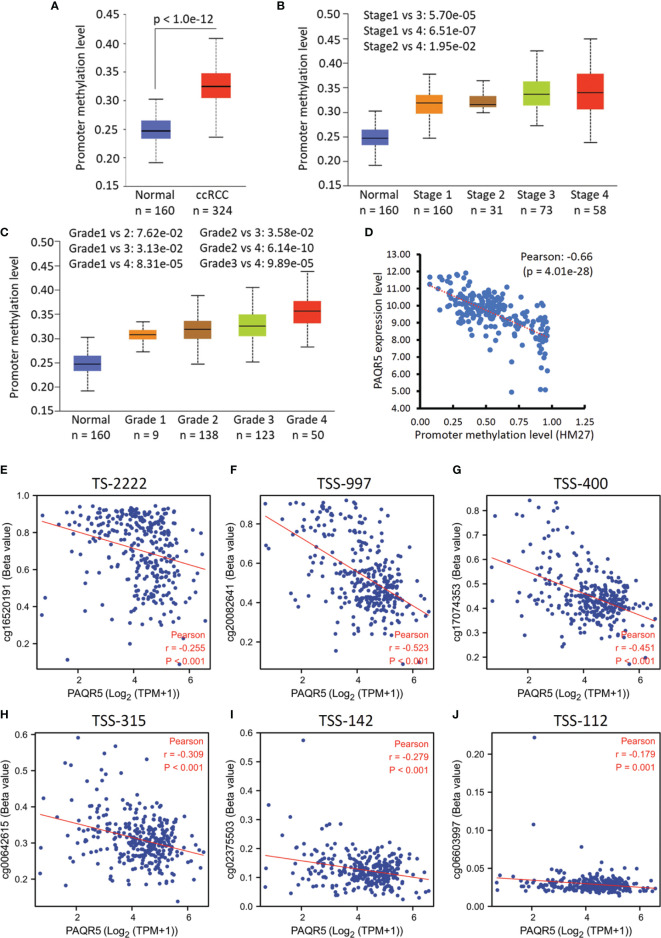
PAQR5 promoter methylation is increased in ccRCC tissues. **(A–C)** Promoter DNA methylation on the PAQR5 gene was analyzed using the TCGA dataset (HM450 BeadChip) derived from ccRCC tissues on the UALCAN platform (29, 30). Tumor stage and cancer grade were set as the variables for multiple group comparison with ANOVA analysis followed by Student *t*-test. Case numbers were listed in each sub-group at the bottom. **(D)** Pearson coefficient analysis was conducted using the TCGA Firehose Legacy RNAseq dataset (HM27 BeadChip) downloaded from the cBioportal platform (case number = 217). **(E–J)** Pearson coefficient analysis was conducted between PAQR5 expression and the DNA methylation levels in a different region upstream of the TSS site using the TCGA RNAseq dataset (HM450 BeadChip).

Since promoter hypermethylation is one of the central mechanisms for gene silencing in human cancers (28), we analyzed the methylation levels of PAQR5 gene promoters based on the TCGA data (29, 30). We first compared PAQR5 promoter methylation levels in ccRCC tissue versus normal kidney tissues. As shown in **Figure 3A**, PAQR5 promoter methylation levels were significantly higher in ccRCC tissues than in normal kidney tissue. The increased promoter methylation was also observed in late-stage (**Figure 3B**) and high-grade tumors (**Figure 3C**). A strong inverse correlation (Person r = -0.66) was observed between PAQR5 expression and promoter methylation in ccRCC tissues (**Figure 3D**). Detailed analysis on different promoter regions revealed that the methylation levels within the -997/-315 region upstream of the transcription starting site (TSS) exhibited the strongest correlation with PAQR5 expression (**Figures 3F–H**) over other regions (**Figures 3E, I, J**), which peaked at the TSS-997 & TSS-400 regions (**Figures 3F, G**). These data indicate that promoter methylation represents a significant mechanism for PAQR5 downregulation in ccRCC tissues, although further mechanistic study is warranted for verification.

### PAQR5 Downregulation Is Associated With Poor Survival Outcomes in ccRCC Patients

**Figure 4:**
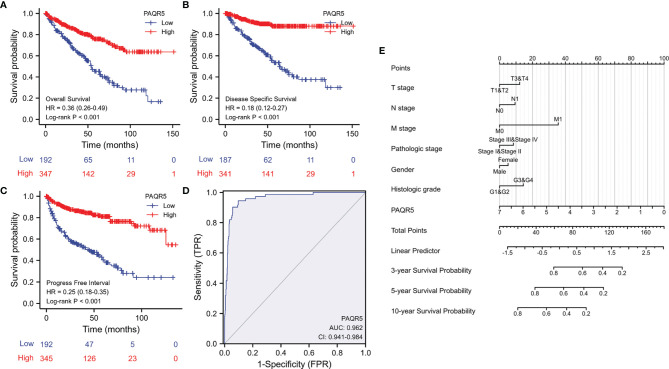
PAQR5 downregulation is associated with poor survival outcomes in ccRCC patients. **(A–C)** Kaplan-Meier survival analysis was conducted in ccRCC patients using the RNAseq data from the TCGA project. Patients were stratified using the minimum p-value approach (26). **(D)** A ROC curve analysis was conducted for overall survival prediction using PAQR5 expression levels in ccRCC patients. **(E)** A nomograph for survival prediction was constructed using the PAQR5 expression levels and the clinicopathological parameters. The nomograph is a calculator tool to predict patient survival rate using clinicopathological data plus the expression levels of biomarker gene. For instance, a male patient had a Grade-1 RCC of T1/N0/M0 at Stage-1, and his PAQR5 expression level was at 3.5. All these parameters will get their nomograph’s points (at the top line). The sum of these points will used to find a linear predictor value, which is applied to find the survival rate. In this case, his total point is 90, then his linear predictor is about 0.75, which indicates his survival rate for 3-year at 60%, 5-year at 40%, and 10-year at 20%.

We next evaluated the impact of PAQR5 expression on patient survival outcomes. Kaplan-Meier survival analysis using the TCGA dataset showed that PAQR5 downregulation had a significant negative impact on patient overall survival outcomes (HR = 0.36), disease-specific survival (HR = 0.18), and progression-free interval (HR = 0.25) (**Figures 4A–C**). Although a univariant regression analysis showed that PAQR5 and traditional clinicopathological parameters were significant prognostic factors for overall survival of patients, multivariate regression analysis showed that only PAQR5 and distal metastasis were significantly associated with overall survival of patients (**Table 2**). A receiver operator characteristic (ROC) curve analysis indicated that PAQR5 expression is a robust prognostic factor with very high specificity and sensitivity (**Figure 4D**). A nomograph was constructed based on the PAQR5 expression data and clinicopathological parameters, which can be used to predict a 3-, 5- or 10-year survival probability for ccRCC patients (**Figure 4E**).

**Table 2:** Prognostic significance of PAQR5 and clinicopathological parameters in ccRCC patients.

Characteristics	Total (N)	HR (95% CI) Univariate analysis	p-value	HR (95% CI) Multivariate analysis	p-value
T stage	539				
T1&T2	349	Reference			
T3&T4	190	3.228 (2.382-4.374)	**<0.001**	1.372 (0.601-3.131)	0.452
N stage	257				
N0	241	Reference			
N1	16	3.453 (1.832-6.508)	**<0.001**	1.291 (0.644-2.590)	0.472
M stage	506				
M0	428	Reference			
M1	78	4.389 (3.212-5.999)	**<0.001**	2.558 (1.520-4.304)	**<0.001**
Pathologic stage	536				
Stage I&Stage II	331	Reference			
Stage III&Stage IV	205	3.946 (2.872-5.423)	**<0.001**	1.273 (0.506-3.206)	0.608
Gender	539				
Female	186	Reference			
Male	353	0.930 (0.682-1.268)	0.648		
Histologic grade	531				
G1&G2	249	Reference			
G3&G4	282	2.702 (1.918-3.807)	<0.001	1.454 (0.868-2.438)	0.155
PAQR5	539	0.636 (0.563-0.718)	<0.001	0.691 (0.576-0.829)	**<0.001**

### TGFB1 Expression Is Inversely Correlated With PAQR5 Expression in ccRCC Tissues

**Figure 5:**
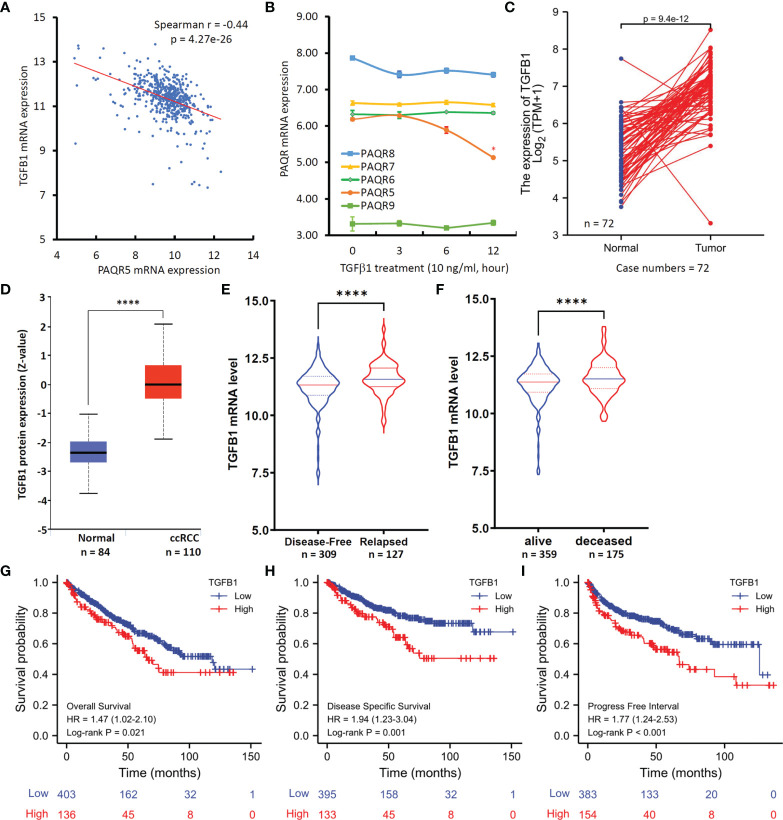
TGFB1 expression inversely correlates with PAQR5 expression in ccRCC tissues. **(A)** Spearman coefficient analysis was conducted using the TCGA-Firehose Legacy RNAseq dataset downloaded from the cBioportal platform (case number = 534). The mRNA levels were presented as log_2_(RSEM+1) values. RSEM: RNA-Seq by Expectation-Maximization. **(B)** Human IOSE cells were treated with TGFβ1 (10 ng/ml) for the indicated time-period. Total RNAs were extracted for gene expression analysis on the Affymetrix Human Genome U133A plus 2.0 GeneChip. The error bar represents the SEM of the mean. The asterisk indicates a significant difference compared to the 0-h control (Student *t*-test, p < 0.05). **(C)** TGFB1 expression levels between case-match pairs of normal kidneys and ccRCC tissues in the TCGA dataset. The p-value was derived from Wilcoxon signed-rank test. **(D)** TGFβ1 protein level in ccRCC and normal kidney tissues using the CPTAC dataset (22). The Quadro-asterisk indicates a significant difference (unpaired *t*-test, p < 0.0001). **(E, F)** TGFB1 expression levels were compared between disease-free *vs.* relapsed or alive *vs.* deceased patients using the TCGA-Firehose Legacy RNAseq dataset downloaded from the cBioportal platform. The Quadro-asterisk indicates a significant difference (unpaired *t*-test, p < 0.0001). **(G–I)** Kaplan-Meier curve analysis was conducted for overall and disease-specific survival, as well as the progression-free interval in ccRCC patients based on TGFB1 expression using the TCGA dataset with a minimum p-value approach (26).

In exploring the potential signaling pathways involved in PAQR5 downregulation, we analyzed the Spearman correlation coefficients between PAQR5 and the entire transcriptome (20020 genes). PAQR5 was negatively correlated with 2563 genes (Spearman r < -0.3) and was positively correlated 1646 genes (Spearman r > 0.3) (**Supplementary Table S3**). Gene enrichment analysis revealed that KEGG pathways of progesterone-mediated oocyte maturation and oocyte meiosis were significantly enriched among those negatively correlated genes (**Supplementary Table S4**), which was in line with PAQR5 function (10, 31). Additional enriched KEGG pathways included ribosome, cell cycle, cancer-related pathways, focal adhesion, and intracellular signal transduction pathways of PI3K-Akt, MAPK, and mTOR. Most interestingly, TGFβ1 signaling pathway was also enriched, and TGFB1 expression was negatively correlated with PAQR5 expression levels (**Figure 5A**).

To understand if TGFβ1 is an upstream regulator for PAQR5 expression, we analyzed gene expression profiles in TGFβ1-treated cells using a microarray dataset (NCBI GDS2795) (24). As shown in **Figure 5B**, PAQR5 expression was significantly reduced in human immortalized ovarian surface epithelial (IOSE) cells after TGFβ1 treatment for up to 12 hours. However, TGFβ1 treatment had no significant effect on other PAQR genes. These data verify TGFβ1 as an upstream suppressor of PAQR5 gene expression.

We then assessed the clinical significance of TGFβ1 expression in ccRCC patients. Compared to normal kidney tissues, TGFB1 expression at the mRNA and protein levels was significantly increased in ccRCC tissues (**Figures 5C, D**). These data are in line with previous reports of increased TGFβ1 plasma level in RCC patients compared to non-malignant controls (32–34). Further analysis revealed that TGFB1 expression was significantly higher in relapsed or deceased ccRCC patients compared to relapse-free or alive patients (**Figures 5E, F**). In addition, higher TGFB1 expression at the mRNA level was significantly associated with poor overall (**Figure 5G**) and disease-specific survival outcomes (**Figure 5H**), as well as shorter progression-free intervals (**Figure 5I**) compared to patients with lower TGFB1 expression. These data are consistent with a previous prognosis study using TGFβ1 protein level as the biomarker for ccRCC patients (35).

## Discussion

In this study, our analysis revealed that the PAQR5 gene was predominantly expressed in normal kidney tissue when compared to other organs, and was significantly downregulated in ccRCC tissues, as assessed at the mRNA and protein levels. PAQR5 downregulation was associated with tumor progression, including tumor stage, cancer grade, lymph node invasion, and distal metastasis. PAQR5 expression was significantly reduced in ccRCC tissues from relapsed or deceased cases. In addition, patients with lower PAQR5 expression had an inferior survival outcome than patients with higher PAQR5 expression. DNA methylation analysis indicated that PAQR5 promoter methylation in ccRCC tissues was significantly higher than in normal kidney tissues and correlated with tumor stage and cancer grade in ccRCC tissues, indicating that DNA methylation could be a potential mechanism for PAQR5 downregulation. In addition, PAQR5 expression was negatively correlated with TGFB1 expression, which was significantly increased in ccRCC tissues and associated with a poor prognosis. TGFβ1 treatment in human cancer cells significantly suppressed PAQR5 expression. As far as the authors are aware, this is the first report about the expression of PAQR genes in human kidney cancers.

Current literature showed that mPRs were involved in cancer progression of the breast, ovarian, prostate, brain, bladder, and uterine (10). Altered expression levels of mPR genes were associated with disease prognosis in breast, endometrial, bladder, and prostate cancers (12–15, 36, 37). Specifically, PAQR6 gene upregulation or copy number gain was associated with an unfavorable prognosis in the prostate, bladder, and endometrial cancers (13, 14, 36). In contrast, an increased expression of PAQR5/8 genes was associated with a favorable prognosis in endometrial cancers (12). This study found that PAQR5 expression was significantly reduced in RCC tissues compared to normal kidney tissues. However, PAQR5 expression correlated considerably with all pathological and clinical parameters, including tumor stage, cancer grade, lymph node invasion, distal metastasis, disease relapse, and survival outcomes only in ccRCC but not in pRCC or ChRCC. These results suggest that PAQR5 potentially plays a tumor-suppressive role in ccRCC development and progression, although further mechanistic study is needed to elucidate PAQR5 function in ccRCC progression.

The individual mPR protein was shown to localize on the plasma membrane and other organelle membranes (10, 38). Immunostaining studies have demonstrated that PAQR7/8 was mainly localized at the plasma membrane, while PAQR5 was localized in the cytoplasm and nucleus in normal endometrium (12). Our results showed that PAQR5 protein was expressed in the cytoplasm of renal tubular cells but not in the glomerulus. In ccRCC tissues, PAQR5 protein staining was primarily lost in the cytoplasm with weak signals at the plasma membrane. We speculate that PAQR5 protein loss might be due to PAQR5 gene downregulation in ccRCC tissues.

The TGFβ1 protein is a multi-functional cytokine, and its role has been implicated in human cancers (39), including enhancement of proliferative and metastatic potential in human RCC cells (40). Increased TGFB1 mRNA or TGFβ1 protein level in cancer tissue or patient plasma was identified as a prognostic factor for rapid progression and poor survival outcome (32–35). Our analysis discovered a strong and inverse correlation between PAQR5 and TGFB1 in ccRCC tissues. Similar to PAQR5, TGFB1 expression was also associated with rapid disease progression and unfavorable survival outcomes. TGFβ1 treatment suppressed PAGR5 expression but did not affect other PAQR genes in human immortalized epithelial cells. It is postulated that increased TGFB1 expression in ccRCC tissues is a potential mediator for PAQR5 downregulation, although further mechanistic study is warranted.

To date, very few reports studied the biological functions of mPR proteins in cancer biology, especially their roles in different types of human cancers after progesterone stimulation. It has been shown that mPR proteins might work either with G-proteins, growth factor receptors, or alone to stimulate diverse intracellular signaling pathways including MAPK, JNK, PI3K, NF-κB modulate cellular function (10). Further studies are needed to understand progesterone-stimulated mPR signaling in renal function or cancer cell behavior.

## Conclusion

PAQR5 expression was significantly reduced in ccRCC tissues compared to normal tissues, as verified at the protein levels. PAQR5 downregulation was significantly associated with clinical and pathological parameters in ccRCC patients, representing a novel prognostic factor of rapid disease progression and poor survival outcome. PAQR5 downregulation was accompanied by increased promoter methylation, indicating a potential mechanism for reduced gene expression. PAQR5 expression was strongly and inversely correlated with TGFB1 expression in ccRCC tissues. TGFβ1 treatment specifically reduced PAQR5 expression, representing a distinct mechanism for PAQR5 modulation.

## Data Availability Statement

The original contributions presented in the study are included in the article/**Supplementary Material**. Further inquiries can be directed to the corresponding authors.

## Author Contributions

RZ and BL designed the study. CT, XY, MY, and BL analyzed the bioinformatics data. WL and SY performed the IHC experiments. BL and QS performed the statistical analysis and generated figures and tables. RZ, QS, and BL drafted the manuscript.

## Conflict of Interest

The authors declare that the research was conducted in the absence of any commercial or financial relationships that could be construed as a potential conflict of interest.

## Publisher’s Note

All claims expressed in this article are solely those of the authors and do not necessarily represent those of their affiliated organizations, or those of the publisher, the editors and the reviewers. Any product that may be evaluated in this article, or claim that may be made by its manufacturer, is not guaranteed or endorsed by the publisher.

## Supplementary Material

Supplementary Figure S1 Gene expression profiles in normal tissues. The cDNA microarray dataset generated from 65 tissue types (19) was analyzed for the gene expression profiles. The figure panels were created on the Oncomine platform. The most predominant tissue types were highlighted in red font.

Supplementary Figure S2 PAQR5 expression in pan-cancer comparison.

Supplementary Table S1 The relationship of PAQR5 expression with clinicopathological parameters in pRCC patients.

Supplementary Table S2 The relationship of PAQR5 expression with clinicopathological parameters in ChRCC patients.

Supplementary Table S3 Spearman correlation analysis of PAQR5 expression in the whole transcriptome.

Supplementary Table S4 Gene-list enrichment analysis of PAQR5-related genes.

## Abbreviations

AR, androgen receptor; BSA, bovine serum albumin; ChRCC, chromophobe RCC; ccRCC, clear cell RCC; CPTAC, clinical proteomic tumor analysis consortium; ER, estrogen receptor; IOSE, immortalized ovarian surface epithelial; KEGG, kyoto encyclopedia of genes and genomes; KICH, kidney chromophobe cell carcinoma; KIRC, kidney renal clear cell carcinoma; KIRP, kidney papillary carcinoma; MAPK, mitogen-activated protein kinase; mPR, membrane-bound progesterone receptors; mTOR, mammalian target of rapamycin; PI3K, phosphoinositide 3-kinase; PAQR, progesterone and adipoQ receptor; PGRMC1/2, progesterone receptor membrane component 1/2; PGR, progesterone receptor; RCC, renal cell carcinoma; pRCC, papillary RCC SEM, standard error of the mean; TGFB1, transforming growth factor beta 1; TCGA, the cancer genome atlas; TBS-T, tris-buffered saline with tween-20.
