# Supplementary figures and images for: PAQR5 Expression Is Suppressed by TGFβ1 and Associated With a Poor Survival Outcome in Renal Clear Cell Carcinoma
Source: Front Oncol. 2022 Jan 20;11:827344. doi: 10.3389/fonc.2021.827344 (PMC8810503; doi:10.3389/fonc.2021.827344)

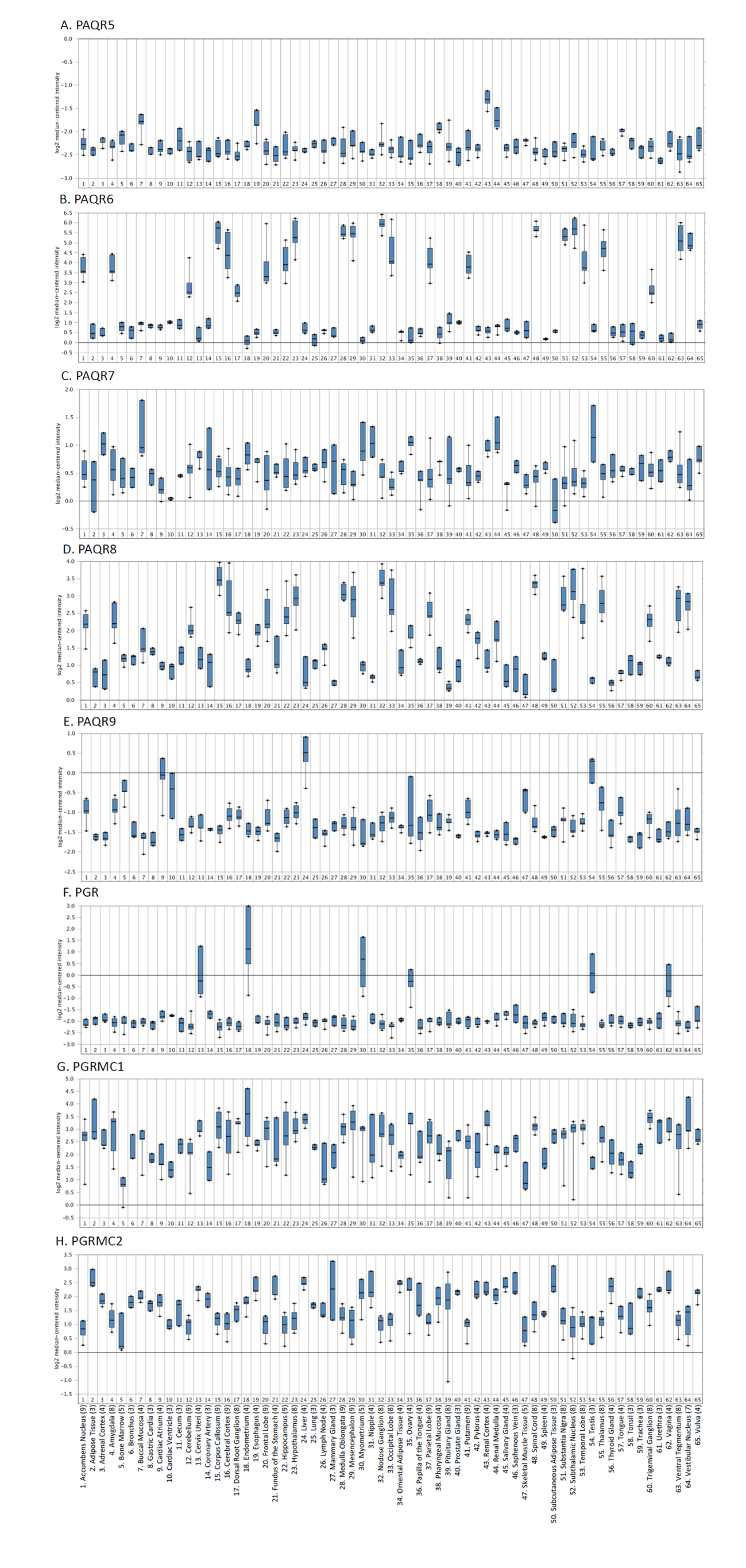

Supplement: Supplementary Figure S1 — Gene expression profiles in normal tissues. The cDNA microarray dataset generated from 65 tissue types (19) was analyzed for the gene expression profiles. The figure panels were created on the Oncomine platform. The most predominant tissue types were highlighted in red font. [file Image_1.tif]

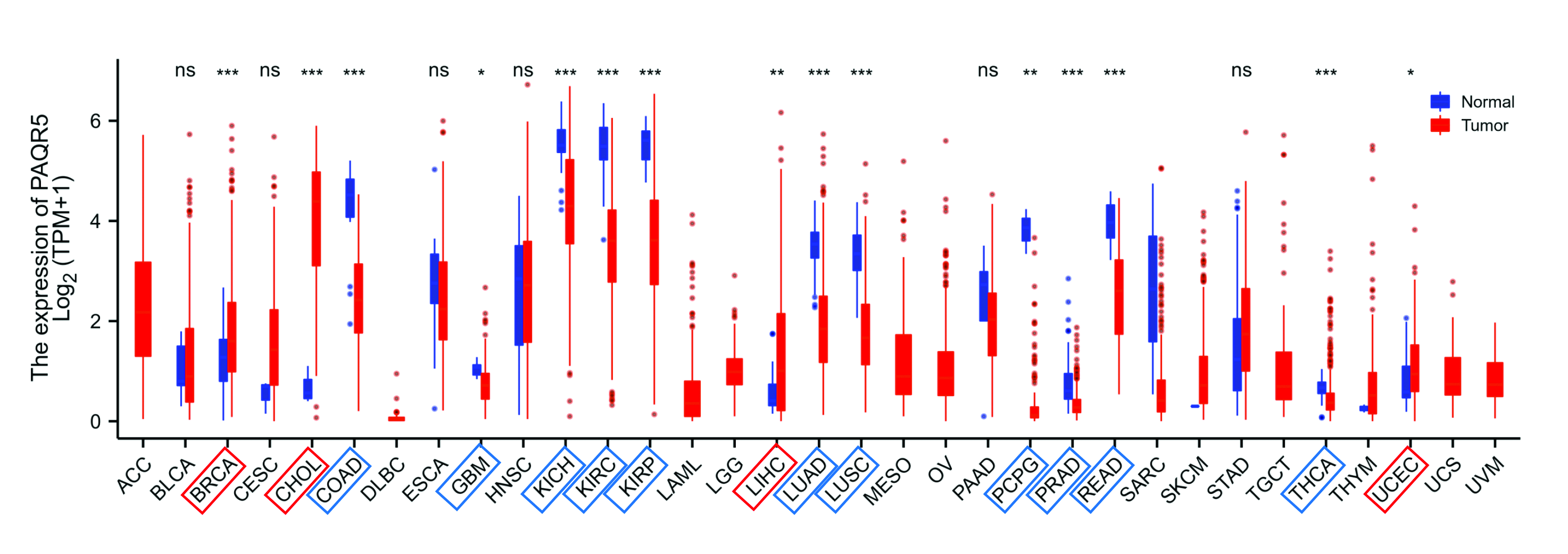

Supplement: Supplementary Figure S2 — PAQR5 expression in pan-cancer comparison. [file Image_2.tif]
